# A Blueprint for Improving the Research Infrastructure of a Pediatric Hospital Medicine Division

**DOI:** 10.7759/cureus.104911

**Published:** 2026-03-09

**Authors:** Gina L Gallizzi, Tamara L Gayle, Anand Gourishankar, Kavita Parikh, Margaret L Rush, Amina Touma, Susan C Walley, Priti Bhansali

**Affiliations:** 1 Pediatric Hospital Medicine, Children's National Hospital, Washington, DC, USA; 2 Pediatric Hospital Medicine, George Washington University School of Medicine and Health Sciences, Washington, DC, USA

**Keywords:** academic medicine, clinical researcher, faculty development, pediatric hospital medicine, research infrastructure

## Abstract

Background: While research barriers in academic medical practice have been identified in prior studies, the facilitators and barriers to research productivity for pediatric hospital medicine (PHM) faculty in the era of board certification are not well described.

Objectives: This study aimed to (1) identify the perceived facilitators and barriers to research productivity among PHM faculty at a large tertiary care children’s hospital, and (2) explore potential differences between fellowship-trained and non-fellowship-trained faculty in their perceptions of the facilitators and barriers to research.

Methodology: A survey was distributed to eligible PHM faculty members from the Division of Pediatric Hospital Medicine of a large tertiary care children’s hospital. The survey, developed using the Bland model of faculty research productivity as a framework, assessed factors related to mentorship, networking, resource utilization, and training. Data were collected over 14 weeks and analyzed using descriptive statistics.

Results: The response rate was 41%, with 28 faculty members responding out of 68 total. Non-clinical time and mentorship emerged as the strongest facilitators. Additional facilitators included supervised health services research and networking. Research barriers included time limitations, burnout, and limited supporting resources. There was no statistically significant difference in answers for the most common barriers comparing the fellowship-trained and non-fellowship-trained groups.

Conclusions: This study identifies key facilitators and barriers to research productivity among PHM faculty, highlighting the importance of mentorship, non-clinical time, and resources. These insights suggest actionable strategies to enhance research productivity. The strategies aim to foster a culture of scholarly productivity within the division and may serve as a model for similar academic PHM divisions.

## Introduction

Pediatric hospital medicine (PHM) is an emerging field with significant potential for academic and scholarly contributions, particularly in research. Research is a critical pillar of academic practice and is vital in furthering a specialty and the associated knowledge base. However, barriers to developing research productivity among academic physicians are well described. Prior survey studies have identified difficulty finding mentors and limited non-clinical time as common barriers for physicians (including PHM physicians) and dentists [1-7].

PHM was recognized as a subspecialty by the American Board of Medical Specialties in 2016 [8]. As a new field, the PHM workforce comprises fellowship-trained and non-fellowship-trained physicians. While scholarship training is an expectation of fellowship [9], both these groups of physicians may have variable experience conducting research. Also, they may experience similar barriers to ongoing research productivity [10]. The field of PHM is broad; clinical practice varies significantly, including care at both community and children’s hospitals, surgical and medical co-management, and care of newborns [11-12]. Forster et al. underscore the importance of capacity building, emphasizing protected time for research, pilot funds, and biostatistical and research coordinator assistance [13]. With the expansion of the field, there is an opportunity for increased academic productivity, but conversely, expanded clinical and administrative roles may limit an individual’s ability to engage in scholarly work. To our knowledge, facilitators of research productivity for PHM faculty have not been described. This study addresses these knowledge gaps related to research in academic PHM practice. 

Our study was aimed at determining the principal perceived facilitators and barriers to conducting research in our division. This was a planned needs assessment informing actionable steps to improve the research infrastructure and support system for faculty in our division. The secondary objective was to investigate potential differences between fellowship-trained faculty and non-fellowship-trained faculty in their perceptions of the facilitators and barriers to research. Any noted differences between these two groups could affect our future steps to increase research support and infrastructure.

This article was previously presented as an abstract at the 2025 Society of Hospital Medicine Converge conference on April 23, 2025.

## Materials and methods

Overview of approach

We surveyed a large PHM division at a single center, which included both fellowship- and non-fellowship-trained physicians, with a mix of instructors, assistant professors, associate professors, and professors. We defined research as scholarship that is performed in a systematic manner, aligns with an investigative framework, is made public and available for peer review, reproducible, and expandable. For our purposes, this included not only traditional research but also work in quality improvement, advocacy, or medical education if it met the proposed definition. Incorporating components of the Bland et al. model of faculty research productivity as a framework, we developed a survey instrument to identify the perceived facilitators and barriers of research productivity in our PHM division [14]. The Bland et al. model uses three domains of characteristics (institutional, individual, and leadership) as inputs for the goal of a productive research organization. This study was given exempt status by our hospital's Institutional Review Board.

Survey development

The survey instrument was informed by prior studies of barriers to research productivity amongst medical professionals and supplemented by a focus group with faculty in our division to identify other factors that facilitate or hinder research productivity unique to PHM as a field or to our specific hospital setting [1-5,14]. The resulting survey draft was reviewed by a survey design expert and modified following six cognitive interviews with PHM faculty from other institutions to achieve validity in the response process. The final survey instrument was developed using REDCap electronic data capture tools [15-16]. It was then piloted with five PHM faculty from various institutions who were different than the cognitive interview group. The wording of the survey explicitly stated that respondents should answer based on their current experience. The survey is included as Appendices A-E.

Sample/site characteristics

Our institution is a large free-standing tertiary care children’s hospital in the Mid-Atlantic region. The division of pediatric hospital medicine additionally staffed five community hospital sites and a sub-acute care facility at the time of survey distribution, with physicians performing various clinical roles across multiple service lines, including newborn medicine, complex care, and general inpatient pediatrics at both community and tertiary care teaching hospitals. Our division is also home to a three-year ACGME-accredited PHM fellowship program. The survey was distributed to 68 attending physicians in the division, including 24 associate or full professors, 26 assistant professors, and 10 instructors. Eight other physicians worked part-time or as needed with the PHM division. Six of the associate or full professors were clinical professors, and two members of the division were on the tenure track.

Our PHM division had established some programs before this study to help develop researchers and a research infrastructure, including a monthly project brainstorming session, a weekly writing accountability group, a pilot grant award program, a health services research (HSR) program, and curated online scholarship resources, including an active projects repository.

Data collection

The survey link was electronically distributed by weekly email for 14 weeks from March 2024 to June 2024 to all 68 eligible physician faculty members. All questions were optional; none were required to be answered to complete the survey. Responses were anonymous. Current PHM fellows and the study investigators were excluded. Part-time and per diem physicians were included, as they may have had unique perspectives on barriers and facilitators of research in our division. 

Data analysis

We used descriptive statistics, such as absolute numbers, percentages, median, and interquartile range (IQR), for categorical and continuous variables as appropriate. Each survey instrument question response was summarized as a horizontal bar graph (%). We assigned values to each response to report a single average response for barriers and facilitators (Exceptional = five points, Significant or Quite = four points, Moderate = three points, Slight = two points, None = one point). Differences in survey responses between fellowship-trained and non-fellowship-trained faculty groups were additionally analyzed using nonparametric tests. The two-sided *P*-value used was less than or equal to 0.05. Data analysis was done using R (version 4.0; R Foundation for Statistical Computing).

## Results

**Figure 1:**
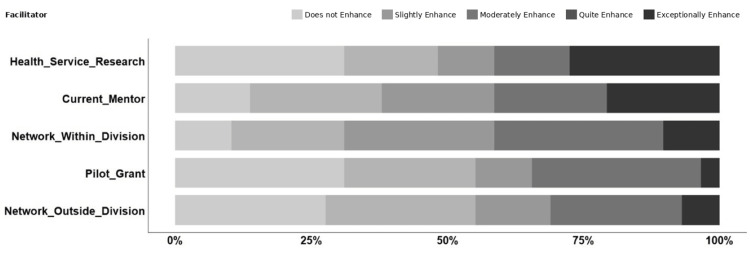
Percentages of all responses to the top five facilitators reported in the survey.

Twenty-eight faculty members completed survey questions about current facilitators and barriers to research. Tables 1-2 show the age and academic rank of respondents, with most respondents aged 40-49 years and holding the rank of assistant professor. We defined the highest-scoring facilitators as those with the most answers of a score of four, *quite helpful*, and a score of five, *exceptionally helpful*, on a Likert scale (with a score of one equating to *does not enhance my research ability at all*). The highest-scoring facilitators were our division’s HSR program, having current mentorship, networking/collaboration within the division, availability of a pilot grant award in the division, and networking outside the PHM division (Figure 1). Self-identified gender or race/ethnicity was not ranked highly as a facilitator by any respondents.

**Table 1:** Academic rank of respondents.

Academic rank	Total number of respondents	Percentage of respondents	Percentage of the eligible population
Instructor	1	4%	16%
Assistant professor	13	54%	43%
Associate/full professor	10	42%	40%

**Table 2:** Survey-reported demographics of respondents.

Reported demographics	Total number of respondents	Percentage of respondents
Hospital Medicine Fellowship		
No	15	60%
Yes	10	40%
Age (years)		
30-34	3	13%
35-39	4	17%
40-44	9	39%
45-49	4	17%
50 or greater	3	13%
Have a research mentor		
No	16	62%
Yes	10	38%

**Figure 2:**
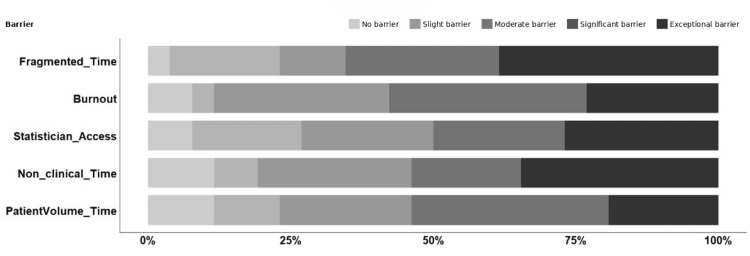
Percentages of all responses to the top five barriers reported in the survey.

We determined the top five barriers using the same approach. Twenty-six responses were provided in the barriers section. The top five barriers were related to time, biostatistician availability, and burnout. The survey included three questions explicitly regarding time: “time due to patient volume,” “limited protected time/non-clinical time,” and “fragmentation of time with other work responsibilities/I don't have large enough chunks of time.” All of these time-related questions were identified in the top five barriers. Fragmented time was identified as the barrier with the most significant and exceptional responses (17, 65%), followed by burnout (15, 58%), having less nonclinical time (14, 54%), time related to higher patient volume (14, 54%), and limited access to a statistician (13, 50%) (Figure 2). We deliberately did not define burnout to allow this domain to be interpreted by the individual. Similar to facilitators, most respondents did not identify gender or race/ethnicity as a barrier.

Tables 3-4 indicate the total and average agreement with the top five facilitators and barriers. Overall, there was more agreement with the top five barriers than with the facilitators.

**Table 3:** Total number of responses and average scores for the top five facilitators of research. The number of respondents who gave each rating to the top five facilitators is displayed. The *Weighted Average* column reflects the total number of responses multiplied by the point value for each rating, divided by the total number of responses within the facilitator category. HSR, health services research

Facilitator	Exceptional (*n*) (5 points)	Quite (*n*) (4 points)	Moderate (*n*) (3 points)	Slight (*n*) (2 points)	Not/None (*n*) (1 point)	Weighted average
HSR training	8	4	3	5	9	2.9
Current mentor	6	6	6	7	4	3.1
Network within the division	3	9	8	6	3	3.1
Pilot grant	1	9	3	7	9	2.52
Network outside the division	2	7	4	8	8	2.55

**Table 4:** Total number of responses and average scores for the top five barriers to research. The number of respondents who gave each rating to the top five barriers is displayed. The *Weighted Average* column reflects the number of total responses multiplied by the point value per rating, divided by the total number of responses for the barrier category.

Barrier	Exceptional (*n*) (5 points)	Significant (*n*) (4 points)	Moderate (*n*) (3 points)	Slight (*n*) (2 points)	Not/None (*n*) (1 point)	Weighted average
Fragmented time	10	7	3	5	1	3.77
Burnout	6	9	8	1	2	3.62
Statistician access	7	6	6	5	2	3.42
Limited non-clinical time	9	5	7	2	3	3.58
Patient volume time	5	9	6	3	3	3.38

The responses of the top five identified barriers and facilitators of those with fellowship training compared with those without fellowship training were not statistically significant (Tables 5-6).

**Table 5:** Association between facilitator and PHM fellowship training status. *Median (IQR). *P*-value was derived using the Mann-Whitney U non-parametric test. IQR, interquartile range; PHM, pediatric hospital medicine

Facilitator	Fellowship*	No fellowship*	95% CI	*P*-value
HSR training	4 (1.3-4.8)	3 (1-5)	-2 to 1	0.97
Current mentor	4 (2.3-4.8)	3 (2-3.8)	-2 to 1	0.23
Network within the division	3 (2-4)	3 (3-4)	-1 to 2	0.34
Pilot grant	2 (1-4)	2.5 (1-4)	-1 to 2	0.79
Network outside the division	4 (2.3-4)	2 (1-3)	-3 to 0	0.07

**Table 6:** Association between barrier and PHM fellowship training status. *Median (IQR). *P*-value was derived using the Mann-Whitney U non-parametric test. IQR, interquartile range; PHM, pediatric hospital medicine

Barrier	Fellowship*	No fellowship*	95% CI	*P*-value
Fragmented time	3.5 (3-5)	4 (3.5-5)	-1 to 2	0.71
Burnout	3 (3-4)	4 (3-4)	-1 to 1	0.66
Statistician access	3 (1.3-3.8)	2 (1-3)	-2 to 1.1	0.38
Limited non-clinical time	3 (2-4)	4 (3-5)	0-2	0.09
Patient volume time	3.5 (2-4)	4 (3-4.5)	0-2	0.22

## Discussion

Our study identified specific actionable facilitators and barriers for research productivity in a large pediatric hospital medicine division. Participants indicated that intra-divisional collaboration/networking and mentorship are the predominant facilitators. Our HSR training program also received a high score. This program was developed by a PHM faculty researcher to provide a mentored group structure for conducting HSR and led to multiple published manuscripts. This program was also anecdotally quite popular within the division, and our results indicate that there is a demand for this type of program with built-in learning and mentorship. D’Arrietta et al. demonstrated that similar research training is motivational and confidence-building for those health professionals with less research experience [6-7]. Mentorship has consistently been shown to help develop an academic career while limited or poor mentorship similarly is identified as a barrier to success [1-7]. Our results indicate that respondents with mentors find that relationship beneficial. While survey respondents did not identify limited mentorship as a significant barrier as time limitations and burnout, most respondents did not report having a research mentor. This suggests a need to establish more intentional mentorship opportunities within our division.

Time-related issues were the most frequent barriers to research productivity, including fragmentation of time, likely related to the nature of clinical responsibilities in our discipline. Opportunities to address this concern may include coaching on how best to maximize productivity by breaking down scholarly tasks into manageable pieces that can be completed in smaller but more frequent time allotments over multiple days [17].

Upon reviewing our findings in the context of the Bland et al. model for a productive research organization, our study team identified factors that limited research in our study fell mostly within the individual and institutional characteristics of productive research organizations [14]. Therefore, to enhance research productivity within the division, the following five steps have been proposed: (1) Develop a division-wide research interest group that focuses on up to two facilitated group projects annually, supported by our research coordinator and a small amount of non-clinical full-time equivalent (FTE) provided for the project lead. This program may create a division environment conducive to research productivity by promoting mentorship, facilitating logistics, and providing dedicated time for project completion. (2) Build scholarship skills within the division by using specific faculty development sessions to provide education on research fundamentals, such as the use of technology and databases, development of specific aims, study design, basic biostatistics, and time management strategies. Supporting professional development in research skills will enable a more equal playing field for division members who may have less prior experience. (3) Provide support to PHM faculty through the creation of a research consult service managed by the division’s research leadership team and research coordinator. This service will provide faculty with assistance in accessing and using resources (i.e., data retrieval, statistical consultations, etc.) as well as building a connection with the research leadership team. (4) Continue ongoing and grow peer/near peer mentorship opportunities to be more purposefully inclusive of faculty members without identified research mentors. Mentorship is a well-known facilitator of research and academic productivity. (5) Promote efforts by our division wellness committee to further probe concerns about burnout that appear to be impacting multiple faculty members in our division. 

We identified multiple limitations of our study. Our survey was distributed to faculty members of one division at one academic center, limiting the generalizability of our findings. The response rate was 41%, which is higher than what is reported for the overall physician response rate for web-based surveys, and within the range of response rates for the Association of Pediatric Program Directors approved surveys of between 21% and 59% for the years 2022-2024 [18-19]. As such, we are unable to determine how the perceptions of facilitators and barriers of research productivity in our sample compare to those of faculty in our division who chose not to complete the survey. However, 28 respondents represent a sizable number of faculty to consider actionable interventions. Associate and full professors were overrepresented in the respondents, while instructors were underrepresented (Table 1). Those with research experience and interest may have been more comfortable with and interested in answering our survey, creating a response bias. We included part-time and per diem attendings in recruitment; these physicians may have been less invested in research and scholarly work and consequently less likely to respond. Finally, this survey does not reflect actual research productivity, as we did not ask about numbers or types of publications.

Despite these limitations, strengths of the study included having a robust approach to our survey design, a varied study team that included members at the assistant, associate, and full professor ranks to help inform the initial research approach, and the identification of actionable items that may improve research productivity within our division. To our knowledge, our study is the first to describe facilitators for research productivity among PHM faculty.

## Conclusions

In conclusion, the identified facilitators and barriers to research productivity in this study will serve as the basis for the next steps to further augment research support and infrastructure in our division. Our findings and approach may also serve as a model for other pediatric hospital medicine divisions seeking to augment their own research productivity. Future studies may explore characteristics of the national landscape of PHM research productivity. Further delineation of the academic physician's perception of time-related barriers may help identify potential structural changes that could facilitate research productivity. Identifying and addressing the research needs of pediatric hospitalist faculty has the potential to improve academic productivity and career satisfaction, ultimately enhancing healthcare delivery for hospitalized children.

## Figures and Tables

**Figure 3:**
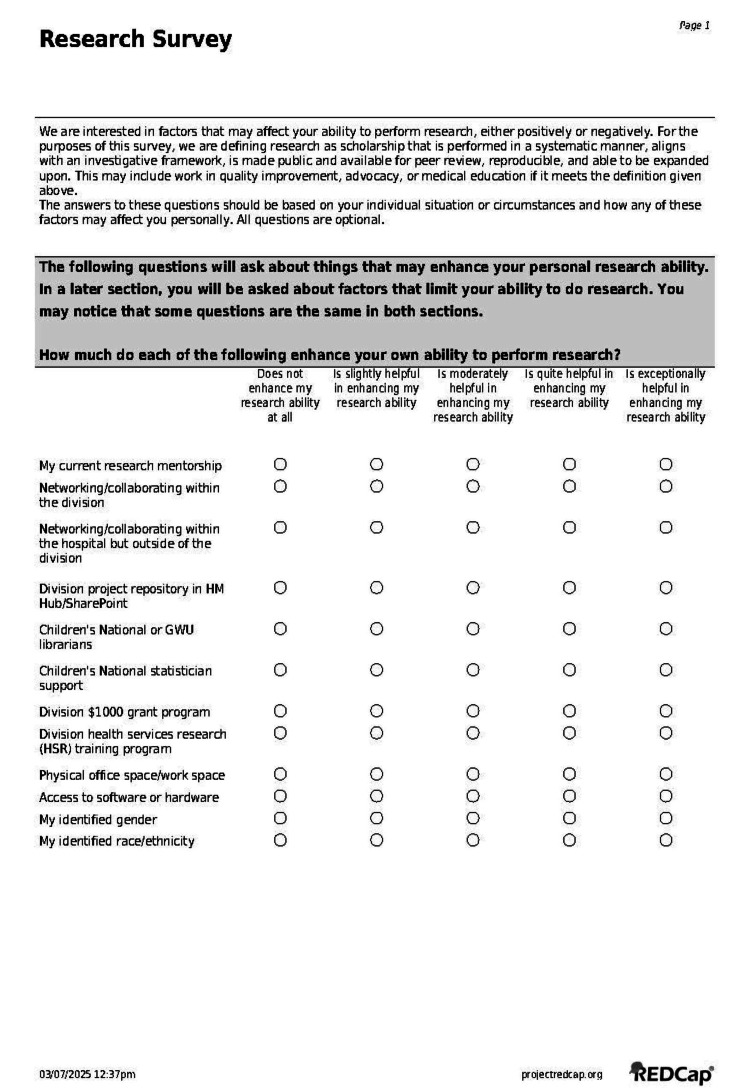
Page 1 of REDCap survey instrument.

**Figure 4:**
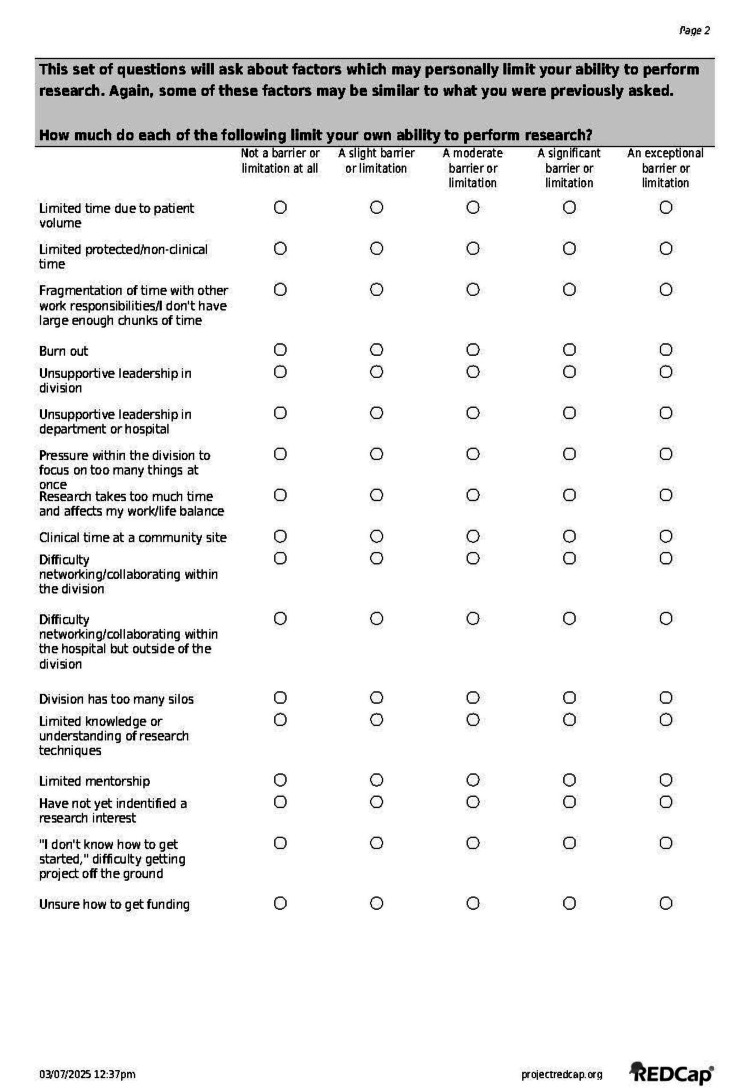
Page 2 of REDCap survey instrument.

**Figure 5:**
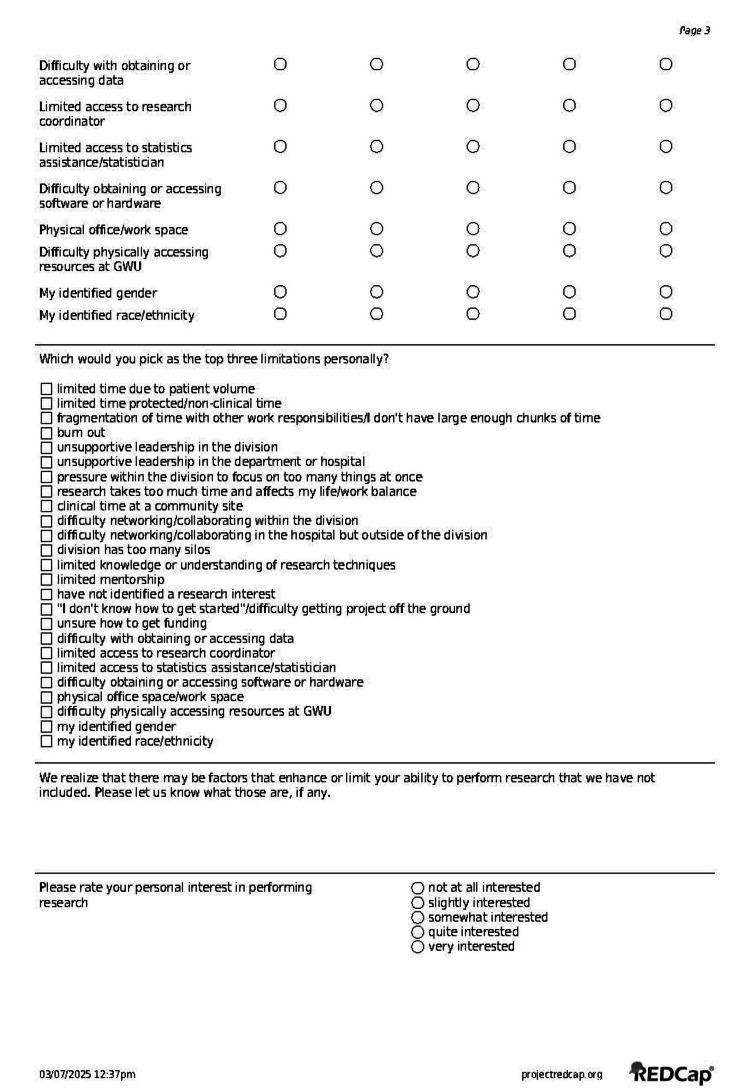
Page 3 of REDCap survey instrument.

**Figure 6:**
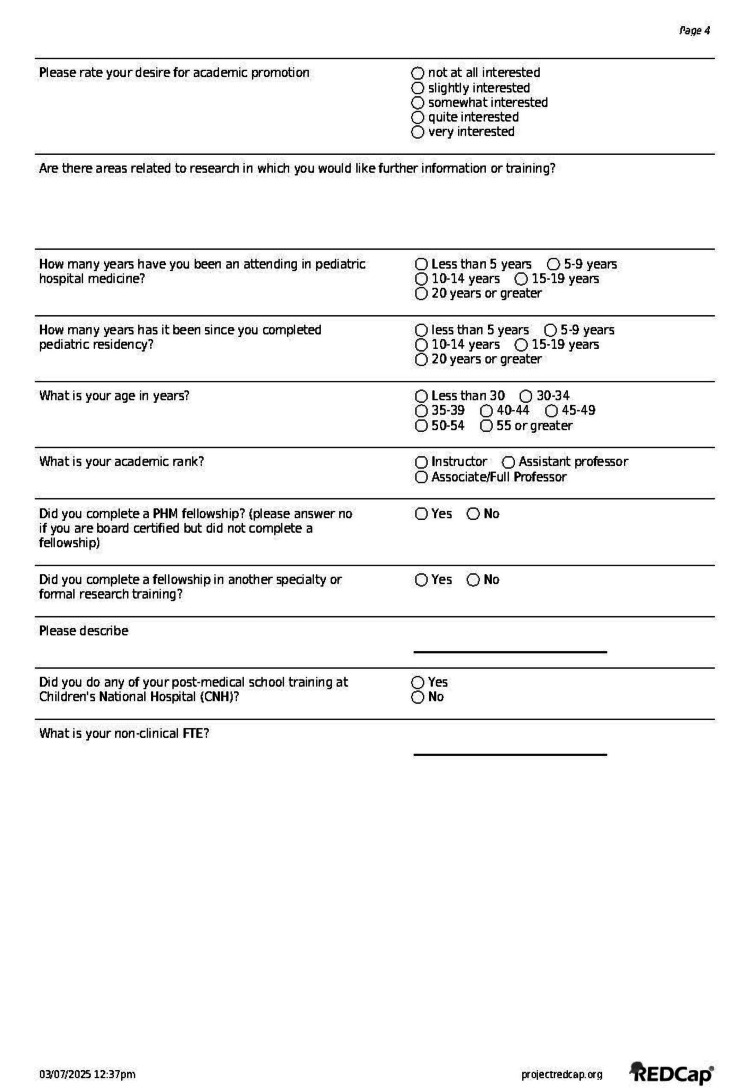
Page 4 of REDCap survey instrument.

**Figure 7:**
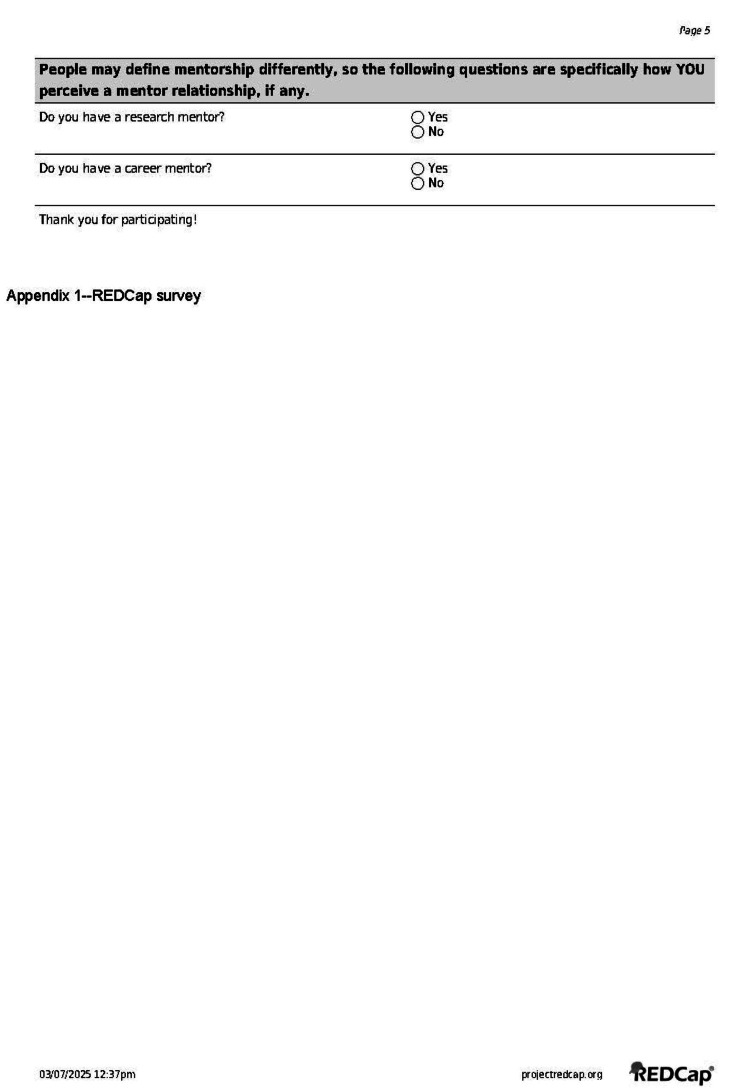
Page 5 of REDCap survey instrument.

## Appendices

Appendix A

Appendix B 

Appendix C 

Appendix D 

Appendix E 
